# Spatial and Temporal Distribution of Di-(2-ethylhexyl) Phthalate in Urban River Sediments

**DOI:** 10.3390/ijerph15102228

**Published:** 2018-10-11

**Authors:** Chih-Feng Chen, Yun-Ru Ju, Yee Cheng Lim, Jih-Hsing Chang, Chiu-Wen Chen, Cheng-Di Dong

**Affiliations:** 1 Department of Marine Environmental Engineering, National Kaohsiung University of Science and Technology, Kaohsiung 81157, Taiwan; dong3762@nkust.edu.tw (C.-F.C.); yrju@nkust.edu.tw (Y.-R.J.); yeecheng@nkust.edu.tw (Y.C.L.); 2 Department of Environmental Engineering and Management, Chaoyang University of Technology, Taichung 41349, Taiwan; changjh@cyut.edu.tw

**Keywords:** di-(2-ethylhexyl) phthalate, DEHP, urban river, seasonal variation, sediments

## Abstract

This study investigated the spatial distribution of di-(2-ethylhexyl) phthalate (DEHP), and its potential biological effects, in the surface sediments that were collected from 10 sites at the Love River during dry and wet seasons. The grain size and organic matter were measured to understand the key factors that affect the distribution of DEHP concentrations in the sediments of Love River. The mean DEHP concentrations in the sediments that were collected during the wet and dry seasons were 28.6 ± 19.5 and 17.8 ± 11.6 mg/kg dry weight, respectively. The highest DEHP concentration was observed in the sediments that were sampled in the vicinity of the estuary. The correlation analysis showed that the grain size and organic matter may play a key role in the DEHP distribution in the sediments during the dry season, whereas the DEHP concentrations in the wet season may be mainly affected by other environmental and hydrological conditions. By a comparison with the sediment quality guidelines, the levels of DEHP in the sediments of Love River were found to have the potential to result in an adverse effect on aquatic benthic organisms. Specifically, during the wet season, wastewater from upstream of Love River is flushed downstream, causing a higher DEHP concentration in the sediments. Future pollution prevention and management objectives should move towards reducing the discharge of upstream wastewater and establishing a complete sewer system to reduce DEHP pollution in the environment.

## 1. Introduction

**Figure 1:**
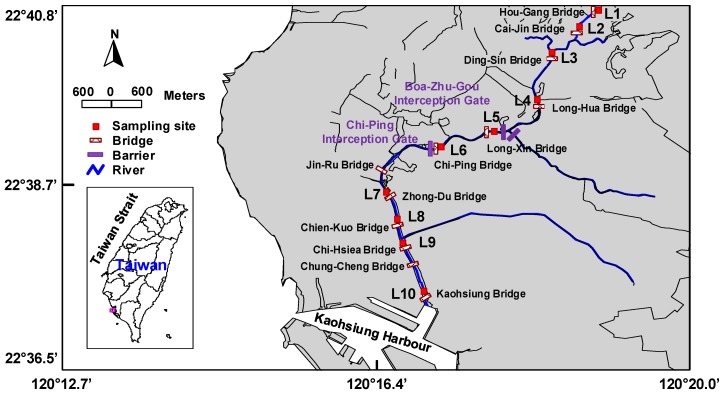
A map of the study area and the locations of the monitoring points.

Kaohsiung City is a highly industrialized city in southern Taiwan with a population of 2.8 million people. Southern Taiwan has a tropical climate; hence, Kaohsiung City has distinct dry and wet seasons [1]. The period from May to September is classified as the wet season, and its rainfall accounts for about 88% of the total annual rainfall [2]. Love River, one of the major rivers in Kaohsiung City, has a length of 16 km and a basin of 62 km^2^. From its main source near Tsao-Gong irrigation, the Love River flows through the downtown region of Kaohsiung City and eventually into Kaohsiung Harbor (Figure 1). About 60% of the total population in Kaohsiung City lives in the regions along the Love River, which include numerous car wash factories, medical institutions, and construction sites. Moreover, the Love River is a canal that receives discharges from domestic, industrial, and farmland wastewater, resulting in an unstable water supply. About 15 drainage canals have been connected to the Love River from upstream to downstream. Since intercepting gates have been set up only in the middle and lower reaches to block the wastewater from entering the drainage canals (Long-Hua Bridge to Hou-Gang Bridge, Figure 1), upstream drainage canals are, therefore, the major pollutant sources [3]. Eventually, pollutants deposit and accumulate in the sediments and may pose potential risks to the local aquatic organisms.

Di-(2-ethylhexyl) phthalate (DEHP) is applied as a plasticizer in the production of plastic polymers, with a usage that accounts for about 50−60% of phthalate ester plasticizers [4,5,6]. Based on reports in the literature, about 2–15 million tons of phthalate ester plasticizers are being produced worldwide annually [7,8,9]. Because DEHP has widespread applications and it weakly binds with plastics, large amounts of it may be released into the environment directly from plastic materials and products. Consequently, DEHP has been commonly found in various environmental media, including air, water, soil, sediment, and even inside living organisms [4,6,10]. DEHP has been reported to have toxic and estrogenic effects on wildlife, and can disrupt the reproduction and developmental systems of several organisms and induce their genetic mutation [11,12,13,14]. DEHP has been identified as a kind of environmental hormone, since it could mislead the ligands of the hormonal system in organisms by its hormone-like structure to interfere with metabolic functions [12]. To protect the environment from the impacts of DEHP, standards and regulations of environmental risk management for DEHP have been established, including the Maximum Contaminant Level (MCL) for drinking water, Environmental Quality Standards (EQS) for seawater and freshwater, Environmental Risk Limits (ERLs) for soil and sediment, and the Minor Adverse Effect Concentration (MAEC) for marine sediments [6]. DEHP, with its hydrophobic nature, is strongly adsorbed on the surface of suspended particles in aquatic environments. DEHP-binding particles deposit at the bottom of an aquatic environment and eventually accumulate in the sediments [15,16]. It is well-known that the sediment acts as a sink that stores the pollutants away from the surrounding matrix, and it could also be a source of pollutants through a re-suspension effect that brings threats to aquatic organisms [10,17,18,19].

For the protection of the aquatic environment and the development of a management strategy, knowledge of DEHP’s distribution in, and its potential ecological effects on, the aquatic sediment is therefore necessary. Nowadays, most studies use a huge amount of experimental data on model organisms combined with various statistical methods to derive sediment quality guidelines (SQGs), which are widely used to assess the potential toxicity effects of pollutants in sediments on aquatic organisms [20]. MacDonald et al. [21] used the weight-of-evidence approach to derive the threshold effect level (TEL) and the probable effect level (PEL) for 34 chemicals and assess the percent incidence of adverse effects within each concentration range. The concentrations of chemicals below the TEL and above the PEL represent the minimal probability and the highest frequency of adverse effects on organisms, respectively [21,22,23]. Moreover, a laboratory toxicological assay incorporating the equilibrium partitioning method was applied to estimate the Maximum Permissible Concentrations (MPCs) and Ecotoxicological Serious Risk Concentrations (SRC_eco_) of DEHP [24]. Therefore, the objectives of this study were to understand the level, spatial and temporal distributions, source, and potential ecological effects of DEHP in the sediments of Love River. This study provides valuable and basic information to help establish a pollution management strategy specifically for the sediments of urban rivers in cities with a high industrialization and population density.

## 2. Materials and Methods

### 2.1. Sample Collection and Analysis

Ten sediment monitoring points (L1–L10) were set up along the Love River from the upstream section to the river’s mouth in October 2011 and July 2012 (Figure 1). The monthly rainfall before the sampling dates of October 2011 and July 2012 was 83.5 and 833.5 mm, respectively (http://www.cwb.gov.tw/). Because of the distinct difference in these rainfall amounts, October and July were classified respectively as the dry season and the wet season in this study. Surface sediment samples were collected from the 10 monitoring points in the dry season (October) and wet season (July) with a 6” × 6” × 6” Ekman Dredge grab sampler. Immediately after collection, the surface sediment samples (0–10 cm) were scooped into amber glass bottles (sealed with a Teflon-lined cap) that had been pre-washed with *n*-hexane and kept in an icebox, and were then transported to the laboratory for analysis.

The sediments were dried with a freeze dryer for 72 h before analysis. The freeze-dried sediments were ground into fine particles using a zirconia mortar and pestle and then screened by a sieve (mesh size = 0.5 mm) [25]. These samples were placed in an amber glass bottle (with a Teflon gasket and a screw cap) that had previously been rinsed with *n*-hexane, and then stored in a freezer at −20 °C. The original fresh sediment was taken to analyze the grain size using a Beckman Coulter LS230 Laser Diffraction Particle Size Analyzer (Beckman Coulter, Inc., Brea, CA, USA) and to measure the organic matter (OM) using the loss-on-ignition (LOI) method. Analytical methods for DEHP (including sample preparation, extraction, cleanup, measurement, and quality control) have been reported in detail previously [26]. Ultrasonic extraction, desulfurization (activated copper), and dewatering (anhydrous sodium sulphate) were done to prepare the samples before the analysis using gas chromatography with mass selective detection using the internal standard (chrysene-d_12_) and surrogate standard (4-terphenyl-d_14_). The detection limit of DEHP is 0.0191mg/kg dw, and the relative percent differences of duplicate samples ranged from 7.6 to 12.1% (*n* = 4). The certified reference materials CRM-143-BNAs-Sandy Loam 1 were analyzed to maintain a quality assurance. The recoveries of the DEHP in the certified reference materials were between 91.3 and 101.8% (*n* = 4) of the certified value.

### 2.2. Data Analysis

A statistical analysis method was employed for the data analysis (e.g., mean, standard deviation, and maximum and minimum values). The obtained data were statistically analyzed by a *t*-test (two-tailed) for the assessment of variation between the dry season and the wet season. Pearson correlation coefficients were used to test the relationship between OM, grain size, and DEHP concentrations in the sediments. SQGs were compared with DEHP concentrations in the sediments to evaluate the ecotoxicity of DEHP [20,27,28]. The threshold effect level (TEL), the probable effect level (PEL), the maximum permissible concentrations (MPCs), the ecotoxicological serious risk concentrations (SRC_eco_), the sediment quality criteria low level (SQC-Low), the SQC upper level (SQC-Up), the environmental risk limits (ERLs), the no observed effect concentration (NOEC), and the predicted environmental concentration (PEC) were also used to compare the measured DEHP concentrations in this study [21,24,29,30,31,32]. Because the values of MPCs have been set on a 10% OM-normalized basis, the original DEHP concentration was divided by the OM content (%) and multiplied by 10 to compare it with the MPCs. These SQGs can be used to classify three ranges of chemical concentrations, including a low concentration range of an unlikely adverse biological effect (e.g., below the TEL or MPC values), a middle concentration range of a possible adverse biological effect (e.g., between the TEL and PEL or the MPC and SRC_eco_ values), and a higher concentration range of probable adverse biological effects (e.g., above the PEL or SRC_eco_ values).

In Taiwan, SQC-Low and SQC-Up are used to assess the quality of sediments and classify sediments into three categories: (1) a pollutant level in the sediments that is lower than SQC-Low means that the sediments have no adverse effect on the ecosystem, (2) between the values of SQC-Up and SQC-Low, the sediments are required to be monitored frequently, and (3) in sediments with a pollutant level that is higher than SQC-Up, sediment remediation must be carried out [32,33]. All analyzed results are respectively shown for the dry season and the wet season and were compared with each other.

## 3. Results and Discussion

### 3.1. Grain Size, OM, and DEHP Content in the Sediments

Table 1 lists the distributions of grain size (sand, silt, and clay), OM, and DEHP content in the sediments collected from the 10 sampling sites at Love River in the dry and wet seasons. The results from the grain size analysis indicate that the sediments were mainly composed of sand and silt. The fine grains were easily taken downstream (L6–L10) by the river water, whereas the coarse grains were readily deposited upstream (L1–L5) due to gravity. Therefore, the upstream sediments were dominated by coarse grains, while the number of fine grains in the sediments gradually increased as the river went down to its lower reaches. During the wet season, the flow of the river is higher than that in the dry season, which could carry or wash the larger-size grains downstream. Hence, the proportion of coarse grains in the downstream sediments is higher in the wet season than in the dry season. However, the aforementioned phenomenon was not found for the temporal (wet season and dry season) and spatial (upstream and downstream) distributions of grain size in the sediments in this study. This may be caused by the interception gates in the middle and upper reaches of Love River that change its hydrology. No obvious spatial–temporal variation was found for the grain size in the sediments. However, overall, the mean proportion of sand in the wet season (55.9 ± 28.7%) was higher than that in the dry season (46.9 ± 39.1%) (*t*-test, *p* = 0.08).

The results from Table 1 show that the sediments contained OM content that ranged from 2.5% to 13.5%. An OM content value higher than 10% was observed at L3, L4, and L10 in the dry season and at L3, L4, L8, L9, and L10 in the wet season, in which higher amounts of pollutants in the sediments may have been adsorbed [34,35]. The mean OM content in the sediment of Love River in the wet season was higher than that in the dry season (*t*-test, *p* < 0.01). During periods of heavy rain in the wet season, the interception gates in Love River are opened, which makes the tributary flow into the main river, and the sewer water stops being collected to prevent the sewage treatment plants from overloading; however, the untreated sewer water will directly enter into Love River, resulting in an increase in the amount of organic matter and nutrients in the river [26,33,36]. In addition, nonpoint-source organic matter might be carried into the Love River with the rainfall runoff during the wet season, and this organic matter is adsorbed onto solid particles and is eventually deposited and accumulates in the sediment.

DEHP was detected in all sediment samples that were collected from Love River with concentrations between 4.2 and 66.7 mg/kg dw, indicating that it is a common pollutant in the river’s environment. Similarly to the trend of OM content, the distributions of mean original DEHP concentration and OM normalized concentration were higher in the wet season (28.6 ± 19.5 and 32.7 ± 17.9 mg/kg dw, respectively) than in the dry season (17.8 ± 11.6 and 26.2 ± 9.8 mg/kg dw, respectively) (*t*-test, *p* < 0.05) (Table 1). The concentrations of DEHP obtained in this research were compared with those published in the literature to understand how serious the problem is for DEHP pollution in Love River. As shown by the data listed in Table 2, Love River has a slightly higher DEPH concentration in its sediment than do other rivers in Taiwan. Compared with other countries, with the exceptions of Yellow River and Yangtze River in China, River Aire in the U.K., and Cross River in Nigeria, Love River’s sediment has a higher DEPH concentration than the sediments of rivers in other countries [34,37,38,39]. According to the report of Sha et al. [34], the highest mean concentration of DEHP was observed in the Luoyang Petrochemical Channel of the Yellow River, which might have been caused by the local industry’s use of a high amount of phthalate esters (PAEs) as raw materials for production and the low flow rate. Sha et al. [34] indicated that the distribution of DEHP in the sediments of the middle and lower reaches of the Yellow River was mainly affected by the inputs of the tributaries, the municipal sewage, and the grain size of the sediment. The discharge from the industrial region was also the main reason for the higher DEHP level in the sediment of part of the Yangtze River [37]. The U.K.’s Land-Ocean Interaction Study monitoring programme identified the occurrence of a range of micro-organic contaminants in the sediments of Humber river, showing that the highest DEHP concentration was found in the sediments of the River Aire, which is characterized by a catchment with a considerable proportion of the urban and industrial activities [38]. Therefore, the higher DEHP concentration in the sediments of Love River might result from the inflow of municipal sewage and industrial wastewater as well as the surface runoff along the river banks [40,41].

### 3.2. Distribution of DEHP in the Sediments of Love River

**Figure 2:**
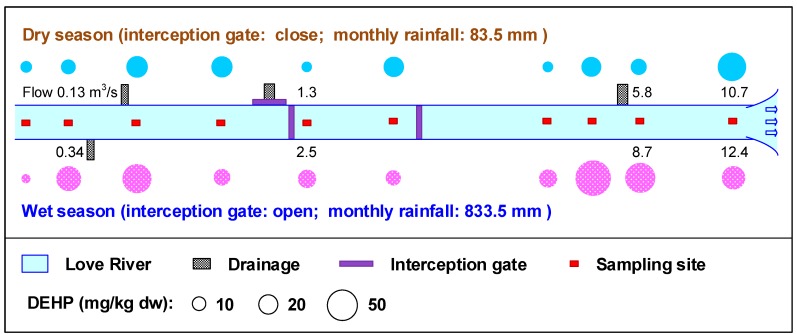
The distribution of DEHP concentrations in the sediments of Love River in the dry season and the wet season.

Figure 2 shows the distributions of DEHP concentrations in the sediments of Love River during the dry season and the wet season. The DEHP concentrations in the sediments ranged from 5.8 to 43.1 mg/kg dw and from 4.2 to 66.7 mg/kg dw during the dry and wet seasons, respectively. The mean DEHP concentration was higher in the wet season (28.6 ± 19.5 mg/kg dw) than in the dry season (17.8 ± 11.6 mg/kg dw) (*t*-test, *p* < 0.01). One of the major reasons for the higher DEHP concentration in the wet season may be the opening of the interception gates in Love River, which makes untreated sewage flow directly into the main channel [56]. Wang et al. [37] analyzed the DEHP concentration of sediment that was collected from the Wuhan section of the Yangtze River in the dry (December to March) and wet (May to September) seasons, and observed that the higher DEHP level was obtained in the wet season. Peijnenburg and Struijs [57] reported that no obvious seasonal variation (spring, summer, and autumn) in the DEHP concentrations in the sediments of rivers was found in the Netherlands, whereas Huang et al. [43] showed that the average DEHP concentration in the 17 rivers of Taiwan (4.1 mg/kg dw) in the dry season (March–April) was significantly (4-fold) higher than that in the wet season (August–October). The highest DEHP concentration in the surface sediment of the Houjing River in southern Taiwan was measured in the dry season (October and December), and Lin et al. [44] speculated that sources of DEHP are still being discharged into the Houjing River. The lack of consistency between the results of these studies and those of the present study could be explained by the different characteristics of each river and its specific environmental and hydrological conditions.

The interception gates in Love River are closed during the dry season, in which case Love River can be divided into three parts; i.e., L1–L4 (the section in front of the Boa-Zhu-Gou Interception Gate), L5–L6 (the section between the Boa-Zhu-Gou Interception Gate and the Chi-Ping Interception Gate), and L7–L10 (the section after the Chi-Ping Interception Gate). In these three sections of Love River, the DEHP concentration in the sediments showed a top-down increasing trend in the dry season (*t*-test, *p* < 0.05). Sediments that were collected behind the location of the interception gates in Love River (L5 and L7) showed relatively lower DEHP concentrations, suggesting that less pollution accumulated in those areas behind the interception gates. The highest DEHP concentration (43.1 mg/kg dw) was found in the vicinity of the estuary (L10). Generally, the fine grain in the sediment is easily carried downstream through a placer mechanism that can separate particles based on gravity during sedimentary processes and lead the fine grains to eventually accumulate in the estuary [33,58]. Given that organic matter tends to adsorb on fine grains, a higher content of pollutants is commonly observed in the estuary area.

In the wet season, the highest concentration of DEHP was observed in the downstream region of Love River (L8–L10), whereas the lowest concentration was discovered in the river’s upper and middle reaches, except for L2 and L3 (*t*-test, *p* = 0.09). Heavy storms during the wet season may intensify the flushing of DEHP-containing surface sediments downstream [44]. The inconsistency of L2 and L3 may be caused by two drainage canals that are located at the interval between L2 and L3 (Figure 2). The sediments in the drainage canals could accumulate a large number of pollutants because the sewer in the upstream region of Love River has not yet been completely constructed and municipal sewage and industrial wastewater from nearby regions directly flow into the drainage canals [3]. The heavy rainfall during the wet season possibly flushes the sediments of the drainage canals into Love River, resulting in the higher concentration of DEHP in the sediments of L2–L3.

**Figure 3:**
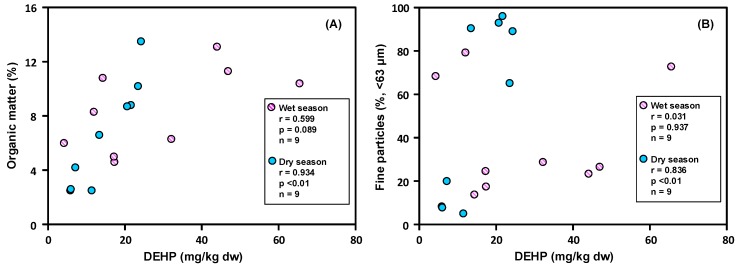
Correlations between DEHP concentrations and (**A**) organic matter as well as (**B**) fine particles in the sediments of Love River during the dry and wet seasons (data from L10 were not include in the correlation analysis, *n* = 9).

The grain size and OM content of sediments are the main factors affecting the level and distribution of hydrophobic organic compounds in sediments [35,40]. In this study, the correlations among the grain size, OM content, and DEHP concentration in the sediments of Love River were examined using a Pearson correlation analysis. According to the preliminary correlation analysis (data not shown), the properties of the sediment at L10 seem to be dissimilar to those of the sediment at other sites. This may be due to the fact that L10 is located in the vicinity of the estuary, which makes it susceptible to the effect of the tides, bringing about transport, mixing, and sedimentation mechanisms at L10 that are quite different from those at the other sampling sites. Therefore, the data from L10 were not included in the correlation analysis in this study. The results of the correlation analysis show that there was no significant correlation between the DEHP concentrations and the proportion of fine grain (*r* = 0.031, *p* > 0.05) and OM content (*r* = 0.599, *p* > 0.05) in the wet season, especially for L4 with the highest OM content and the lowest DEHP concentration (Figure 3). It might be the case that the dominant factors that determine the distribution of DEHP concentration in the sediment during the wet season are not fine grain and OM content but other factors, such as transport, mixing, and sedimentation mechanisms and the composition of sources [10,20,47,59]. During the dry season, the DEHP concentration in the sediments had a significantly positive correlation with OM content (*r* = 0.934, *p* < 0.01) and the proportion of fine grain (*r* = 0.836, *p* < 0.01). The results suggest that both organic matter and grain size play a critical role in the DEHP distribution in sediments in the common case [6,37,51,60,61].

### 3.3. Evaluation of Potential Ecological Effects

DEHP is reported to cause bioaccumulation, toxicity, and hormonal imbalance in aquatic organisms, affecting the reproduction and development functions of these organisms as well as even inducing genetic aberrations [11,12,13,62]. Because of DEHP’s low water solubility, high organic carbon–water partition coefficient (K_oc_) value, and hydrophobicity, it tends to be adsorbed on suspended particles and eventually accumulates in sediments. Therefore, it is necessary to understand the hazardous effects of a sediment’s DEHP level on benthic organisms. However, it is hard to clarify the toxic and hazardous effects of DEHP on aquatic organisms that live in contaminated sediments due to the unimaginably complex contaminants in the sediments [30]. For this reason, this study evaluated the potential ecological effects of DEHP in the sediments of Love River on a benthic habitat through a comparison with the established SQGs [1,10,61].

**Figure 4:**
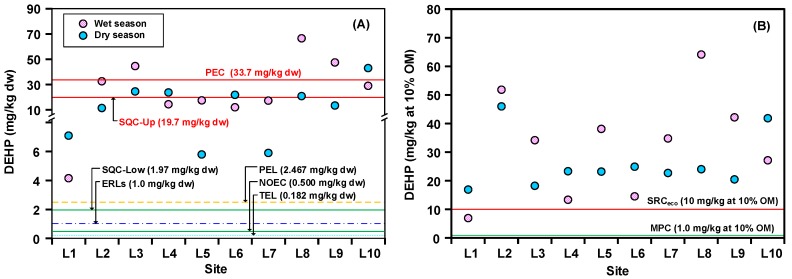
Comparison of the (**A**) original and (**B**) 10% OM-adjusted DEHP concentrations with sediment quality guidelines (SQLs) at 10 monitoring sites during the dry season and the wet season. ERLs, environmental risk limits; PEL, probable effect level; NOEC, no observed effect concentration; TEL, threshold effect level; SRC_eco_, ecotoxicological serious risk concentrations; MPC, maximum permissible concentration.

During the dry season, the DEHP concentrations of sediments collected at all sites exceeded the values of TEL (0.182 mg/kg dw), NOCE (0.500 mg/kg dw), ERLs (1.0 mg/kg dw), SQC-Low (1.97 mg/kg dw), PEL (2.467 mg/kg dw), MPC (1.0 mg/kg at 10% OM), and SRC_eco_ (10 mg/kg at 10% OM), and, specifically, were 2–27 times higher than the PEL (Figure 4). These results indicate that the levels of DEPH in the sediments of Love River may have an adverse impact on benthic organisms. Additionally, sediment DEHP concentrations in the sites along the Boa-Zhu-Gou Interception Gate (L3−L4) and the Chi-Ping Interception Gate (L6) as well as those in the downstream sites were higher than SQC-Up (19.7 mg/kg dw), in which the DEHP level in L10 was even higher than the PEC (33.7 mg/kg dw). Similar to the dry season, the DEHP concentrations in all sediment samples were higher than the PEL, and they were also higher that SRC_eco_, with the exception of L1 (Figure 4). Furthermore, the DEHP concentrations in the sediments that were collected from upstream L2–L3 and downstream L8–L10 were higher than SQC-Up (19.7 mg/kg dw), and, particularly, the DEHP concentrations in the sediment samples from L3, L8, and L9 exceeded the PEC (33.7 mg/kg dw). In summary, based on the comparison with the SQGs, it is possible that the DEHP levels in the sediments of Love River will result in an adverse effect on aquatic benthic organisms regardless of whether it is the dry season or the wet season. There is a need to carry out an ecological risk assessment to acknowledge the hazard that DEHP represents to ecological systems and evaluate whether remediation is necessary, particularly for those parts of the Love River with a DEHP concentration that is higher than the value of SQC-Up. According to the presented results, concern must be raised particularly in the case of sediments in the downstream regions during the wet season because the interception gates in the middle reach during this season are open, which will flush a large number of pollutants to the downstream and estuary area where they will eventually accumulate.

## 4. Conclusions

The DEHP concentrations in the surface sediments of Love River were between 4.2 and 66.7 mg/kg dw. As a result of the opening of the interception gates in the middle section of Love River during the wet season and the significant volume of untreated wastewater flowing into Love River, the mean DEHP concentration in the sediments in the wet season (28.6 ± 19.5 mg/kg dw) was found to be higher than that in the dry season (17.8 ± 11.6 mg/kg dw) (*t*-test, *p* < 0.01). The spatial distribution of DEHP showed that the higher concentration was found in sediments near the interception gates and the estuary area in the dry season (*t*-test, *p* < 0.05), and in the upstream and downstream regions during the wet season. These results suggest that the major factors affecting the spatial distribution of DEHP concentration in the sediments of Love River were not only the municipal sewage and industrial wastewater but also the effect of the drainage canals of the river. The results of evaluating the potential ecological effects showed that the DEHP levels in all sediment samples that were collected from Love River exceeded the PEL, indicating that the environments of Love River may pose a potential ecological risk, especially in the downstream regions. The assessment that was performed in this study further suggested that effective control and management strategies for DEHP in the Love River need to be established and executed to improve the quality of sediments and protect the aquatic organisms in the river basin from the environmental DEHP.

## Figures and Tables

**Table 1:** The distribution of grain size, organic matter (OM) content, and DEHP levels in the sediments of Love River during the dry season and the wet season.

Site		Clay (<2 μm) (%)	Silt (2–63 μm) (%)	Sand (>63 μm) (%)	Organic Matter (%)	DEHP (mg/kg dw)	DEHP (mg/kg dw at 10% OM)
Dry season (flow rate: 0.13−10.7 m^3^/s)							
L1	Hou-Gang Bridge	4.8	15.2	80.0	4.2	7.1	16.9
L2	Cai-Jin Bridge	1.3	3.8	94.9	2.5	11.5	46.0
L3	Ding-Sin Bridge	3.8	85.3	10.9	13.5	24.6	18.2
L4	Long-Hua Bridge	9.6	55.6	34.8	10.2	23.8	23.3
L5	Long-Xin Bridge	1.8	6.5	91.7	2.5	5.8	23.2
L6	Chi-Ping Bridge	22.3	73.8	3.9	8.8	21.9	24.9
L7	Zhong-Du Bridge	1.7	6.1	92.2	2.6	5.9	22.7
L8	Chien-Kuo Bridge	20.0	73.0	7.0	8.7	20.9	24.0
L9	Chi-Hsiea Bridge	21.3	69.2	9.5	6.6	13.5	20.5
L10	Kaohsiung Bridge	11.8	44.5	43.7	10.3	43.1	41.8
Minimum–Maximum		1.3−22.3	3.8−85.3	3.9−94.9	2.5−13.5	5.8−43.1	16.9−46.0
Mean ± Standard deviation		9.8 ± 8.6	43.3 ± 32.5	46.9 ± 39.1	7.0 ± 3.9	17.8 ± 11.6	26.2 ± 9.8
Wet season (flow rate: 0.34−12.4 m^3^/s)							
L1	Hou-Gang Bridge	15.7	52.7	31.6	6.0	4.2	6.9
L2	Cai-Jin Bridge	4.6	24.2	71.2	6.3	32.6	51.8
L3	Ding-Sin Bridge	5.0	18.4	76.6	13.1	44.8	34.2
L4	Long-Hua Bridge	3.9	9.9	86.2	10.8	14.4	13.4
L5	Long-Xin Bridge	4.0	13.5	82.5	4.6	17.5	38.1
L6	Chi-Ping Bridge	13.3	66.0	20.7	8.3	12.1	14.5
L7	Zhong-Du Bridge	4.9	19.7	75.4	5.0	17.4	34.8
L8	Chien-Kuo Bridge	12.1	60.7	27.2	10.4	66.7	64.1
L9	Chi-Hsiea Bridge	4.7	21.9	73.4	11.3	47.7	42.2
L10	Kaohsiung Bridge	15.4	70.7	13.9	10.7	29.1	27.2
Minimum–Maximum		3.9−15.7	9.9−70.7	13.9−86.2	4.6−13.1	4.2−66.7	6.9−64.1
Mean ± Standard deviation		8.4 ± 5.1	35.8 ± 23.8	55.9 ± 28.7	8.7 ± 3.0	28.6 ± 19.5	32.7 ± 17.9

**Table 2:** Comparisons of the DEHP concentrations in the sediments of Love River with those of rivers in other regions.

Region	DEHP (mg/kg dw)	References
Love River, Taiwan	4.2–66.7	This study
Zhonggang, Keya, Erren, Gaoping, Donggang, Danshui Rivers, Taiwan	0.5–23.9	[42]
17 principal rivers in Taiwan	ND ^1^–46.5	[43]
Houjing River, Taiwan	0.10–20.22	[44]
Qiantang River, China	ND ^1^–0.131	[45]
Middle and lower Yellow River, China	5.35–258.5	[34]
Yangtze River (Wuhan section), China	48.0–221.4	[37]
Qiantang River, China	0.365–6.24	[18]
Pearl River, China	0.415–29.5	[10]
Songhua River, China	0.227–0.567	[46]
Jiulong River, China	0.007–0.394	[15]
Gomti River, India	ND ^1^–0.324	[47]
Kaveri River, India	0.278	[48]
Klang River, Malaysia	0.49–15.0	[49]
Sembrong River, Malaysia	2.07–7.50	[50]
Furu River, Japan	1.0–2.0	[4]
Jinzu River, Japan	0.020–0.300	[51]
Oyabe River, Japan	0.02–1.800	[51]
Turano river, Italy	0.0032–0.4873	[52]
River Trent, U.K.	0.84–31.0	[38]
River Aire, U.K.	7.89–115	[38]
Brandenburg and Berlin, Germany	0.21–8.44	[53]
Ogun River, Nigeria	0.020–0.820	[17]
Cross River System, Nigeria	1.97–86.76	[39]
Nzhelele River, Mutshindudi River, Dzwerani River, Lotanyanda River, Xikundu River, Mutale River, Luvuvhu River, Dzindi River, South Africa	0.02–1.12	[54]
Jukskei River, South Africa (Summer)	0.00654–3.66	[55]

^1^ ND: not detectable.
